# Chondrolysis of the Hip following Septic Arthritis: A Rare Complication of Magnetic Resonance Arthrography

**DOI:** 10.1155/2013/840681

**Published:** 2013-02-24

**Authors:** Barak Haviv, Rafael Thein, Alon Burg, Snir Heller, Shlomo Bronak, Steven Velkes

**Affiliations:** ^1^Arthroscopy and Sports Injuries Unit, Hasharon Hospital, Rabin Medical Center, Petach-Tikva 49372, Israel; ^2^Orthopedic Department, Sackler Faculty of Medicine, Tel-Aviv University, Tel-Aviv 69978, Israel; ^3^Department of Orthopedic Surgery, Beilinson Hospital, Rabin Medical Center, Petach-Tikva 49100, Israel

## Abstract

Magnetic resonance arthrography (MRA) is commonly used to detect labral tears of the hip. Complications of MRA are unusual and include minor reactions such as chemical synovitis and urticaria. This paper presents a rapidly progressive chondrolysis of the hip in a young patient after arthrography. The patient had suffered from acute septic arthritis and was treated by emergent arthroscopic surgery followed by appropriate antibiotics. At 18 months of followup, there were no signs of active infection but evidence of joint chondrolysis. Magnetic resonance arthrography (MRA) of the hip is an invasive procedure and should therefore be recommended judiciously. Post-MRA pain is common but often mild and temporary, while post-MRA joint infection is rare; nevertheless, severe joint pain and limitation should raise suspicion for septic hip.

## 1. Introduction

Hip arthroscopy has become a popular procedure to treat various pathologies about the hip joint [1]. Hence, magnetic resonance arthrography (MRA) of the hip is often required prior to surgery in order to delineate intra-articular lesions [2]. Arthrography is considered a safe procedure and may cause pain and anxiety but rarely a major complication such as infection [3]. This case report presents an unusual complication of acute septic hip arthritis caused by arthrography and treated by arthroscopic synovectomy and lavage. 

## 2. Case Report

**Figure 1:**
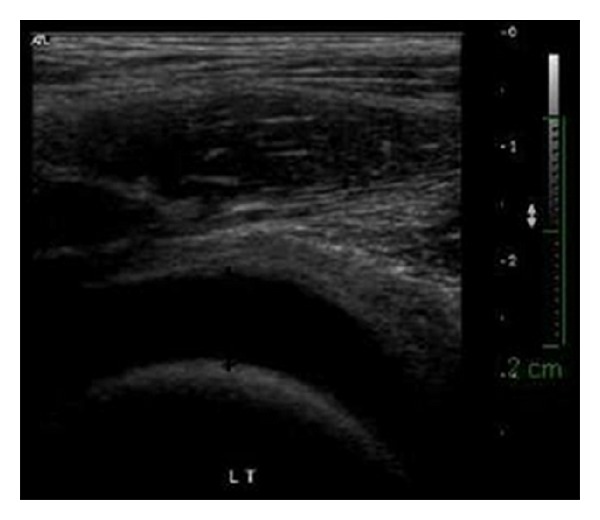
Ultrasound of patient's left hip at the emergency room showing distention of the hip joint due to increased fluid level.

**Figure 2:**
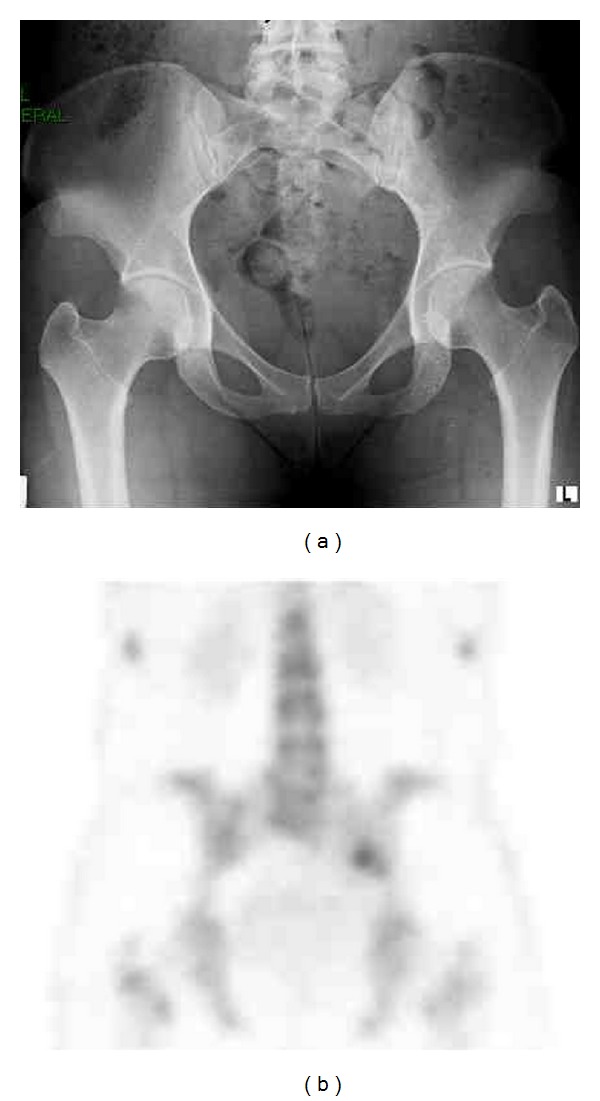
Imaging of the patient at 18 months after surgery: (a) pelvic X-ray shows mild narrowing of the left hip joint and (b) Tc-99m bone scan shows mild uptake at the left sacroiliac joint.

A diagnostic magnetic resonance arthrography (MRA) was ordered from a healthy 35-year-old female with clinical suspicion of femoroacetabular impingement in her left hip. MRA with gadolinium demonstrated an intact labrum and cartilage, unremarkable bone marrow signals, and a small amount of fluid at the origin of the rectus femoris from the anterior inferior iliac spine. Four days later, she arrived at the emergency room because of excruciating left hip pain. She could neither bear weight on her left leg nor move her left hip. On arrival, her body temperature was 37°, the white blood count (WBC) 8.95 K/micl, erythrocyte sedimentation rate (ESR) 80 mm/1 hr, and C-reactive protein (CRP) 24 mg/dL. Ultrasound- (US-) guided hip arthrocentesis retrieved 30 CC of purulent fluid (Figure 1). Fluid analysis was indicative of infection. An emergent 3 portal hip arthroscopic guided by fluoroscopy was performed, as described by Kim et al. [4]. Synovectomy and debridement of granulation tissue was performed by utilizing a motorized shaver followed by a high volume of saline (12 L) joint lavage. The labrum and cartilage appeared normal. No postoperative drains were used. Cultures yielded a positive *Streptococcus* viridans result which was treated by intravenous Ceftriaxone 2 gm/d. During her stay in the hospital, she remained afebrile, pain decreased, and hip range of motion improved. She was discharged at postoperative day 12. Intravenous antibiotics were given for a period of 6 weeks. Nonweight bearing with crutches and full range of motion (ROM) were prescribed for 6 weeks. Sequential inflammatory markers normalized at 3 weeks postsurgery (Table 1). No further surgery was needed. At 18 months of followup, the patient remained in pain with muscle atrophy, limp, and limited range of motion. There was no increased uptake about the hip on Tc-99m bone scan. Plain radiography showed narrowing of the joint space (Figure 2) suggestive of chondrolysis.

## 3. Discussion

This case demonstrates a rare complication of acute septic arthritis of the hip following magnetic resonance arthrography (MRA). Septic arthritis of the hip can be treated successfully with an early arthroscopic intervention. The literature is consistent and provides excellent outcomes for this procedure, but it is limited to one Level II randomized controlled trial and several small Level IV case series [1, 4, 5]. The patient was treated by urgent surgical debridement and irrigation followed by antibiotics to control the infection; however, she remained in considerable pain and difficulties in daily living activities. Her latest postoperative radiographs showed narrowing of the joint space suggestive of chondrolysis similar to previous reports on septic coxarthrosis [5]. 

Several studies have evaluated postarthrographic pain after direct MRA in different joints. Giaconi et al. [6] reported on postarthrographic pain in 20 out of 26 hips (77%) that started the day after injection and resolved over 2-3 days. Other than pain, there were no other reported complications and specifically no cases of septic arthritis. Saupe et al. [7] evaluated 285 postarthrographic hips. Pain was most pronounced 4 hours after MR arthrography and disappeared within 1 week afterwards. No signs of joint infection were found in any of the patients. The pain may be due to joint distention or an inflammatory response developed by the patient in response to a direct chemical irritation by the injected contrast material [8, 9]. 

Two large questionnaire-based retrospective studies have described complications after arthrography [3, 10]. The most recent study by Hugo et al. [10] included 262,000 arthrograms, of which there were approximately 13,300 MR arthrograms. The total complication rate was 3.6%, of which 0.03% were considered severe. Minor reactions included chemical synovitis, vagal reaction, and urticaria. Among severe reactions (overall 75 cases), there were 29 cases of septic arthritis. In another study, by Newberg et al. [3], the risk of joint infection after intra-articular contrast media administration was three per 126 000 cases (0.003%). 

## 4. Conclusions

Post-MRA pain is common but often mild and temporary, while post-MRA joint infection is rare; nevertheless, severe joint pain and limitation should raise suspicion for septic hip. Clinical impression together with high levels of ESR and CRP are suggestive, while US-guided joint aspiration can confirm the diagnosis. Infection should be treated aggressively in order to avoid sepsis and minimize joint damage. Arthrography of the hip is an invasive procedure and should therefore be recommended judiciously. 

## Figures and Tables

**Table 1:** Sequential inflammatory markers of the patient (high levels are indicated in bold).

DATE	CRP (mg/dL)	WBC (K/micl)	ESR (mm/1 hr)
On arrival	**23.91**	8.95	**80**
Postoperative day 6	**12.69**	5.5	**93**
Postoperative day 9	**8.99**	6.9	**74**
Postoperative day 12	**6.3**	8.6	
Postoperative day 13	**5.3**	4.3	14
Postoperative day 23	0.3	5.1	18
Postoperative day 37	0.1	4.0	6

CRP: C-reactive protein; WBC: white blood count; ESR: erythrocyte sedimentation rate.

## References

[1] Stevens MS, Legay DA, Glazebrook MA, Amirault D (2010). The evidence for hip arthroscopy: grading the current indications. *Arthroscopy*.

[2] Osinski T, Malfair D, Steinbach L (2006). Magnetic resonance arthrography. *Orthopedic Clinics of North America*.

[3] Newberg AH, Munn CS, Robbins AH (1985). Complications of arthrography. *Radiology*.

[4] Kim SJ, Choi NH, Ko SH, Linton JA, Park HW (2003). Arthroscopic treatment of septic arthritis of the hip. *Clinical Orthopaedics and Related Research*.

[5] Yamamoto Y, Ide T, Hachisuka N, Maekawa S, Akamatsu N (2001). Arthroscopic surgery for septic arthritis of the hip joint in 4 adults. *Arthroscopy*.

[6] Giaconi JC, Link TM, Vail TP (2011). Morbidity of direct MR arthrography. *American Journal of Roentgenology*.

[7] Saupe N, Zanetti M, Pfirrmann CWA, Wels T, Schwenke C, Hodler J (2009). Pain and other side effects after MR arthrography: prospective evaluation in 1085 patients. *Radiology*.

[8] Hall FM, Rosenthal DI, Goldberg RP, Wyshak G (1981). Morbidity from shoulder arthrography: etiology, incidence, and prevention. *American Journal of Roentgenology*.

[9] Kose N, Inan U, Baycu C, Omeroglu H, Seber S (2007). Effects of intraarticular contrast media on synovial membrane and cartilage: an electron microscopic evaluation in rabbit knees. *Saudi Medical Journal*.

[10] Hugo PC, Newberg AH, Newman JS, Wetzner SM (1998). Complications of arthrography. *Seminars in Musculoskeletal Radiology*.

